# Mucosal Inflammatory Memory in Chronic Rhinosinusitis

**DOI:** 10.3390/cells13231947

**Published:** 2024-11-23

**Authors:** Min-Seok Koo, Sungmin Moon, Min-Seok Rha

**Affiliations:** Department of Otorhinolaryngology, Yonsei University College of Medicine, Seoul 03722, Republic of Korea; kooms97@yuhs.ac (M.-S.K.); kikin90@yuhs.ac (S.M.)

**Keywords:** chronic rhinosinusitis, inflammatory memory, mucosal immunity, trained immunity

## Abstract

Recent advancements in medical management, endoscopic sinus surgery, and biologics have significantly improved outcomes for patients with chronic rhinosinusitis (CRS). However, long-term recurrence is frequently observed following endoscopic sinus surgery, with symptoms worsening after biologics are discontinued. Consequently, refractory or recurrent CRS remains a significant challenge, causing a substantial healthcare burden. In this review, we provide current insights into mucosal inflammatory memory, a potential mechanism leading to CRS recurrence. Given that both immune and non-immune cells in the sinonasal mucosa play critical roles in the pathophysiology of CRS, a deeper understanding of the mechanisms underlying mucosal inflammatory memory in various cellular components of sinonasal tissue could aid in the management of refractory CRS. We describe and discuss the latest knowledge regarding the novel concept of inflammatory memory, including both adaptive immune memory and trained immunity. Additionally, we summarize the pathogenic memory features of the sinonasal mucosa cellular components in the context of CRS.

## 1. Introduction

Chronic rhinosinusitis (CRS) is an inflammatory disease of the nasal cavity and paranasal sinus that lasts more than 12 weeks. The major symptoms of CRS include nasal congestion, nasal discharge, facial pain or pressure, and smell loss, which significantly impairs the quality of life [1,2]. CRS is estimated to affect approximately 5.5–28% of the global populations [1,3] and causes a high socioeconomic burden, with over a million surgical procedures performed annually worldwide [2,4,5].

CRS is conventionally categorized into two subtypes based on the presence of nasal polyps (NPs): CRS without NPs (CRSsNP) and with NPs (CRSwNP) [1,3]. About one-third of patients with CRS have NPs [6], typically exhibiting more severe symptoms and a higher recurrent rate than in the case of CRSsNP. However, since the mid-2010s, the concept of endotyping has emerged to address the heterogeneity of CRS and to develop targeted therapies [7]. Currently, CRS is considered a highly heterogeneous disorder and classified into two or three inflammatory endotypes based on the immunological context in sinonasal mucosa: type 2 and non-type 2 endotypes [8]. Type 2 CRS is characterized by eosinophilic inflammation and predominant type 2 immune responses, including increased frequencies of T helper (Th) 2 cells, type 2 innate lymphocytes (ILC2s), and mast cells in sinonasal mucosa with high expression levels of type 2 cytokines, including IL-4, IL-5, and IL-13 [9,10,11,12]. Non-type 2 CRS exhibits marked type 1 and/or type 3 immune response, occasionally involving considerable neutrophil infiltration in the sinonasal tissue. This endotype is related to the increased Th1 and Th17 cells with high expression levels of IFN-γ, TNF, and IL-17A [11,13,14,15]. In this context, the treatment paradigm for CRS has transitioned from a physical ventilation and drainage paradigm to the ‘mucosal concept’, which focuses on endotyping to enable precision treatment strategies for each patient [16].

Currently, appropriate medical management combined with endoscopic sinus surgery (ESS) is the standard treatment for CRS. Additionally, the advent of biologics has significantly improved treatment outcomes for patients with CRS [17]. However, a study showed that the majority of patients who underwent ESS experienced disease recurrence, and a fraction of them required revision surgery [18]. Additionally, although biologics effectively reduced NP size and symptom severity, symptoms and nasal polyp scores worsened after discontinuation [19]. Therefore, refractory or recurrent CRS remains a significant issue, contributing to a substantial healthcare burden and negatively impacting patients’ quality of life. Patients with severe type 2 CRS frequently exhibit recurrence as well as more severe clinical symptoms. Meanwhile, poor responsiveness to corticosteroid therapy and the lack of validated biologics make recalcitrant non-type 2 CRS difficult to treat [20,21]. As a result, patients with severe non-type 2 CRS have no treatment options other than revision surgery if the disease recurs. 

As described above, the treatment paradigm of CRS has shifted to focus on mucosal inflammation. In this concept, mucosal inflammatory memory is suggested as a key mechanism underlying recalcitrant and recurrent CRS when external insults encounter the sinonasal epithelium. Therefore, a better understanding of mucosal inflammatory memory in the context of CRS would provide critical insights into the development of novel therapeutic strategies for recalcitrant CRS. Specifically, the mechanism underlying the maintenance and induction of pathogenic memory cells in the nasal mucosa is a topic of research interest. Recently, numerous studies have significantly advanced our understanding of inflammatory memory in tissues.

In this context, a narrative literature review on mucosal inflammatory memory in CRS was conducted. The search strategy was to search for the latest and most relevant articles written in English. PubMed was used as a source for all articles until November 2024. The keywords used in the literature search were as follows: ‘Chronic rhinosinusitis’, ‘Nasal polyps’, ‘Memory T cells’, ‘Th2’, ‘Th17’, ‘Inflammatory memory’, ‘Trained immunity’, ‘Immunopathology’, ‘Epigenetic reprogramming’, ‘Allergic inflammation’. All authors independently explored and collected all articles. In case of conflicting opinions, the corresponding author (M.-S.R.) determined whether to add or remove the articles for further analysis.

In the current review, we describe the basic characteristics of mucosal inflammatory memory, ranging from adaptive immune memory to innate immune memory. We also summarize previous studies that have shed light on immunological memory in CRS. Additionally, we discuss newly discovered cellular and molecular mechanisms that regulate inflammatory memory in both immune and non-immune cells. Finally, we share our perspective on therapeutic strategies targeting mucosal inflammatory memory to alleviate CRS.

## 2. T Cell Memory in CRS

### 2.1. An Overview of T Cell Memory

The immune system primarily consists of antigen-specific adaptive immunity and nonspecific innate immunity. T cells are major components of the adaptive immune system and key players in cellular immunity. The cardinal features of T cells are antigen specificity and immune memory. They typically express a clonally derived antigen receptor known as the T cell receptor (TCR) that confers antigen specificity. Upon encountering an antigen, naïve T cells carrying antigen-specific TCRs are activated and differentiate into effector T cells [22]. These effector T cells then move into the site of infection or inflammation to perform their effector functions. After the antigen is eradicated, T cell responses diminish. After the contraction phase, a subset of effector T cells become long-lasting memory T cells capable of rapidly exerting effector functions upon re-encounter with an antigen [22]. Memory T cell subsets include central memory T (T_CM_) cells, effector memory T (T_EM_) cells, terminally differentiated effector memory T (T_EMRA_) cells, and the recently identified tissue-resident memory T (T_RM_) cells. T_RM_ cells are a subset that resides in peripheral tissues [23,24,25]. T_RM_ cells can exist in various tissues, including the skin [26], lung [27], intestine [28], brain [29], reproductive tract [30], spleen [31], lymph nodes [31], and bone marrow [23,32]. T_RM_ cells are commonly characterized by high expression of CD69, CD103, CD49a, and CXCR6, as well as activation markers such as CD38 and PD-1. In particular, CD69 expression is a key indicator of T_RM_ cells in non-lymphoid organs [33] because it inhibits S1PR1-mediated T cell emigration from lymphoid organs [33,34,35]. Additionally, as CD103 is an epithelial cell-binding αEβ7 integrin that interacts with E-cadherin [23], CD103^+^ T_RM_ cells are enriched in epithelial tissues. T_RM_ cells provide long-term, localized, rapid protection and immunosurveillance in barrier tissues as sentinels [36,37,38]. However, several studies have suggested that T_RM_ cells can also be pathogenic in chronic inflammatory conditions [39], such as inflammatory bowel disease [40] and glomerulonephritis [41]. Collectively, T_RM_ cells exhibit functional differences based on the local immune microenvironment and disease state. Thus, determining whether these cells are protective or pathogenic and understanding how they are regulated may be crucial for developing effective T_RM_ cell-oriented treatment strategies for chronic inflammatory diseases.

### 2.2. Altered Phenotypes of T Cells and Pathogenic T Cell Memory in CRS

Type 2 CRS typically involves significant infiltration of eosinophils in nasal tissue [42], along with increased expression of type 2 cytokines and local eosinophil-recruiting factors [3,8,43]. Upon exposure to external stimuli, nasal epithelial cells secrete epithelial-derived cytokines, such as IL-25, IL-33, and thymic stromal lymphopoietin (TSLP), which elicit type 2 immune responses [8]. In the adaptive immune system, Th2 cells are major producers of type 2 cytokines [10,44]. In particular, a subpopulation of memory Th2 cells highly expresses ST2, the receptor for IL-33, and produces IL-5 and amphiregulin upon IL-33 stimulation [45]. These ST2^hi^ memory Th2 cells, referred to as memory-type pathogenic Th2 cells, contribute to the pathogenesis of diverse type 2 inflammatory diseases by potently producing type 2 cytokines [44,46]. Memory pathogenic Th2 cells exhibit elevated expression of ST2, IL-17RB, and CRTH2 [46].

Previous studies have revealed that Th2 cells are enriched in eosinophilic CRSwNP compared with controls and non-eosinophilic CRSwNP [47,48]. Additionally, Th2 cells expressing IL-17RB and ST2 were exclusively observed in NP tissue, but not in control nasal tissue or peripheral blood [49]. These cells exhibit high responsiveness to IL-25 and IL-33 with enhanced production of IL-5 and IL-13. Our group demonstrated staphylococcal superantigen-related expansion of TCR Vβ5.1^+^CD4^+^ T cells exhibiting Th2 phenotypes in non-asthmatic patients with CRSwNP [50]. These cells expressed high levels of CRTH2, IL-17RB, and ST2, and their frequencies positively correlated with disease extent [50]. Furthermore, in vitro treatment with dupilumab reduced proliferation and GATA3 upregulation in CD4^+^ T cells upon IL-4 stimulation, suggesting that dupilumab may inhibit the expansion of memory pathogenic Th2 cells in patients with CRS. A recent single-cell RNA sequencing (scRNA-seq) analysis of NP CD4^+^ T cells uncovered cellular heterogeneity within the Th2 cell population [51]. In the study, most CD4^+^ T cells were memory T cells, and a fraction of Th2 cells expressed CRTH2, whereas a subset of CD109^+^CRTH2^−^ Th2 cells produced the immunosuppressive cytokine IL-10. A very recent study has identified a progenitor subset of Th2 cells that co-express *TCF7* and *LEF1*, exhibiting self-renewal capacity and the potential to differentiate into effector cells [52]. These findings suggest that these memory progenitor cells may play a role in maintaining type 2 inflammation. Further studies with a large number of patients are required to investigate their associations with clinical features, including symptom severity and treatment outcomes.

**Figure 1:**
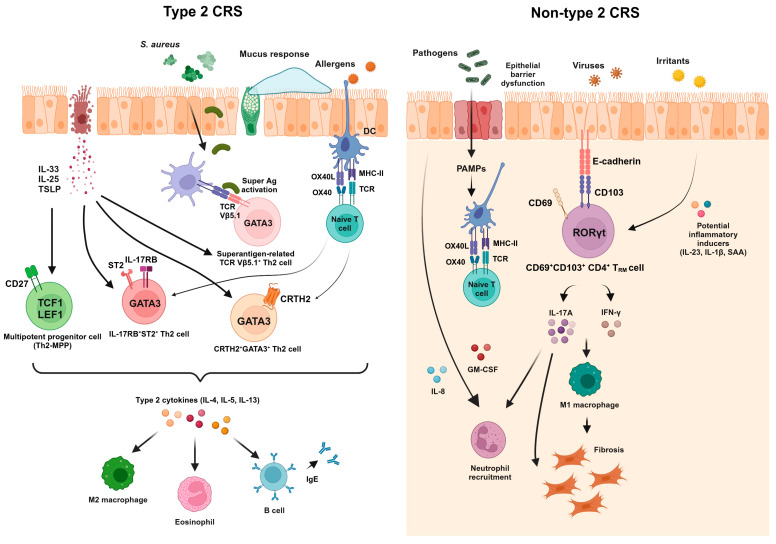
Pathogenic memory T cells and their interactions with other immune cells in the nasal tissue of type 2 and non-type 2 CRS. Phenotypes of memory T cells and their interactions with other immune cells in CRS are depicted across inflammatory endotypes. This figure was created in Created with BioRender.

In the nasal tissue, the majority of CD4^+^ and CD8^+^ T cells are CCR7^−^CD45RA^−^ effector memory T cells with few naïve T cells, regardless of the disease status [53]. Additionally, nasal CD4^+^ and CD8^+^ T_RM_ cells exhibiting activated phenotypes are enriched in the nasal tissue. Given that CD103^−^CD4^+^ T_RM_ cells produce various type 2 cytokines in the lungs of mice upon repeated exposure to *Aspergillus fumigatus* [54], memory pathogenic Th2 cells may be located in the nasal tissue as CD4^+^ T_RM_ cells and contribute to the pathogenesis of allergic airway inflammation. Further studies are required to investigate tissue-resident features of memory pathogenic Th2 cells. Non-type 2 CRS is characterized by an increased proportion of Th1 and Th17 cells, indicating dominant type 1 and/or 3 immune responses [8]. Notably, our group showed that the frequency of nasal CD103-expressing CD4^+^ T_RM_ cells was significantly higher in non-eosinophilic CRS compared with the control and eosinophilic CRS [53]. These T cells displayed a higher expression of Th17-related markers, including CCR6 and RORγt, and possessed robust IL-17A-producing capacities. Given that the Lund–Mackay CT score was correlated with the frequency of CD69^+^CD103^+^ nasal CD4^+^ T_RM_ cells and their capacity to produce IL-17A, the regulation of these cells may offer novel treatment options for non-eosinophilic CRS. Figure 1 presents the phenotypes and effector functions of pathogenic T cells and their interactions with other cells within the nasal tissue of CRS across inflammatory endotypes. In summary, the key characteristics of T_RM_ cells—tissue residency and memory features—may predispose these cells to be critical players in refractory CRS. Remnant pathogenic T_RM_ cells in the nasal tissue, even after treatment, may contribute to recurrence upon stimulation with inhaled insults. Therefore, regulatory targets for pathogenic nasal T_RM_ cells in CRS should be further investigated.

## 3. B Cell Memory in CRS

### 3.1. Memory B Cells and Antibody-Secreting Cells

Memory B cells last long term and differentiate into antibody-secreting cells (ASCs) when re-exposed to an antigen [55]. Upon re-exposure to an antigen, memory B cells enter the germinal center and undergo additional affinity maturation [56]. Antibodies produced by terminally differentiated plasma cells contribute to protection against reinfection [57]. The close interaction between antigen-presenting cells, memory follicular helper T (Tfh) cells, and memory B cells enables vigorous memory responses.

In recent years, several studies have identified a specific subset of memory B cells, known as tissue-resident memory B (B_RM_), localized in peripheral tissues [58,59,60,61,62]. Lung B_RM_ cells exhibit high expression levels of markers related to tissue residency (CXCR3, CCR6, and CD69) and low expression levels of CCR7, SELL, S1PR1, and KLF2 [62]. Similarly, the majority of gut CD27^+^ memory B cells express CD69 in humans [61]. These findings suggest that B_RM_ cells exhibit a tissue-residency signature of T_RM_ cells. However, the key regulators and transcriptional programs driving B_RM_ cell differentiation remain to be elucidated. Furthermore, the memory features of pathogenic B cells in the context of chronic inflammation are not yet well understood.

### 3.2. Changes in Phenotypes and Functions of B Cells and ASCs in CRS

Alterations in B cells, notably the expansion of activated B cells and ASCs, are observed in CRS. A previous study using flow cytometry analysis demonstrated that both B cells and plasma cells are enriched in NPs compared with nasal tissue from control individuals [63]. Additionally, the frequency of CD19^+^CD27^+^CD38^hi^ plasmablasts was significantly increased in NPs [64]. In the nasal tissue of patients with CRSsNP, the number of IgD^+^CD19^+^CD38^bright^ plasmablasts increased [65]. A recent scRNA-seq analysis of CD19^+^ and CD19^−^ ASCs from NPs of patients with allergic fungal rhinosinusitis further revealed that CD19^+^ ASCs were highly enriched in NPs [66].

Accumulating evidence shows that B cells become activated to produce antibodies in the nasal tissue of CRSwNP. Expression levels of germline transcripts, which serve as markers for cells undergoing class-switch recombination, for IgG, IgA, and IgE are increased in NP tissues [63,67,68]. Consistent with this, the accumulation of antibodies of all isotypes was observed in NP tissue [63,67]. A previous study using ELISpot assays and ex vivo cultures showed that NP-derived B cells exhibited more potent producing capacities for IgG, IgA, and IgE compared with tonsillar B cells [64]. Notably, increased IgE production is frequently observed in eosinophilic NPs. IgD-activated mast cells promote IgE production and eosinophilic inflammation in patients with CRSwNP [69].

Several recent studies have reported the existence of tertiary lymphoid structures or ectopic lymphoid tissues in a fraction of patients with CRS and suggested their contribution to the immunopathogenesis of CRS [68,70,71]. Lau et al. identified the formation of tertiary lymphoid organs in 37% of NP tissue from patients with CRSwNP, but not in tissue from control individuals [70]. The presence of tertiary lymphoid organs was suggested to be associated with upregulation of inflammatory genes [71]. Another study described that ectopic lymphoid tissues were linked to local immunoglobulin production, thereby contributing to the development of CRSwNP [68]. The frequency of IL-4-producing BCL-6^+^CD4^+^ Tfh cells in ectopic lymphoid tissues correlated with local IgE levels in eosinophilic NPs.

However, direct evidence supporting the detrimental roles of B cells and ASCs in CRS is still limited, and it would be of interest to identify inflammatory endotypes that are closely associated with B cells and ASCs. Additionally, the regulatory mechanisms governing B cells and immunoglobulin production in NP tissue remain unclear. Further research is needed to identify memory phenotypes and tissue-resident features of pathogenic B cells, as well as their interaction with other immune cells. A better understanding of memory B cells in the nasal tissue will serve as a basis for the development of next-generation therapeutics against CRS.

## 4. Epithelial Stem Cell Memory in CRS

### 4.1. Basic Characteristics and Physiological Roles of Nasal Epithelial Cells

Epithelial cells in the sinonasal mucosa act as the frontline barrier against inhaled pathogens and actively defend against infections at their earliest stages. Over the past several decades, the perspective on the nasal epithelium has evolved from considering it as a physical barrier to an immunologic organ. Nasal epithelial cells are crucial to host defense, interacting with other immune cells to maintain homeostasis [72].

The nasal epithelium has traditionally been described as consisting of basal cells, suprabasal cells, goblet cells, and ciliated and nonciliated columnar cells [73]. Basal cells serve as progenitor cells for the airway epithelium, capable of proliferating in response to injury and differentiating into other cell types. The airway is primarily lined by ciliated and secretory cells, which facilitate mucociliary clearance, a process that removes inhaled particulates and irritants. Mucociliary clearance is regulated by mucus secretion and the coordinated ciliary beating [8]. Coordinated ciliary beating moves airway mucus toward the pharynx where it can be cleared by swallowing [72]. Goblet cells produce mucins, which play a critical role in preventing pathogen entry into the epithelium. In recent research, scRNA-seq analysis further delineated the cellular heterogeneity of the nasal epithelium, identifying novel cell populations with distinct molecular characteristics. Ruiz Garcia et al. clustered human nasal epithelial cells into several subsets: cycling basal, noncycling basal, suprabasal, club, goblet, deuterosomal, and multiciliated cells [74]. Additionally, ionocytes and myoepithelial cells were identified as rare cell subsets [75].

The key structures of the physical epithelial barrier consist of tight junctions, adherens junctions, and desmosomes/hemidesmosomes [4]. Tight junctions include ZO-1, occludin, claudins, and junctional adhesion molecule 1 proteins. The innate epithelial immunity of the nasal epithelium is complemented by functional receptors that recognize pathogen epitopes and elicit rapid immune responses. Pattern-recognition receptors detect a broad array of conserved microbial ligands, referred to as pathogen-associated molecular patterns (PAMPs) [76]. Upon PAMP recognition, Toll-like receptors regulate the release of antimicrobial peptides from epithelial cells.

### 4.2. Impaired Epithelial Barrier Function and Dysregulated Epithelial Repair in CRS

Harmful environmental factors, including respiratory pathogens, allergens, and pollutants, disrupt tight junctions. Barrier disruption results in increased exposure to foreign antigens and stimuli, triggering an immune response. Several studies have demonstrated that impaired barrier function is a key feature of CRS, characterized by disruption of tight junctions and increased ion permeability. Additionally, acquired ciliary dysfunction is frequently observed in CRS [77].

The disruption of the epithelial barrier initiates repair processes and activates the epithelial–mesenchymal transition (EMT). The EMT is a process by which epithelial cells lose their cell polarity and acquire migratory and invasive phenotypes to become mesenchymal stem cells [78]. Chronic EMT is a hallmark of CRS. In CRS, the EMT is marked by the loss of tight junction proteins and a decrease in E-cadherin, cytokeratins, and vimentin. The loss of junction proteins leads to detachment from the basement membrane, loss of polarity, and increased cell division to repair the injury. At the same time, basal cells differentiate into mesenchymal cells that produce various extracellular matrix proteins, including desmin, fibronectin, tenascin, laminin, and collagens. Several factors, such as TGF-α, oncostatin M, epiregulin, and HIF-1α, are associated with the EMT in CRS [4,79].

### 4.3. Pro-Inflammatory Functions of Epithelial Cells in CRS

Epithelial cells also exert pro-inflammatory and immune-modulating functions by producing cytokines. In response to inhaled antigens, epithelial cells induce migration of dendritic cells (DCs) into the epithelium through CCL20 [80]. Subsequently, epithelial cells regulate DC-mediated control of Th cell differentiation by producing TSLP. Exposure to environmental factors, such as fungal allergens like *Alternaria* extracts, triggers the secretion of type 2 epithelial cell-derived cytokines [81,82]. *Staphylococcus aureus* infection also induces the secretion of TSLP and IL-33 from epithelial cells [83]. Additionally, protease-containing allergens and type 2 cytokines stimulate TSLP production by epithelial cells [84,85]. Particulate matter exposure is associated with the secretion of inflammatory cytokines (IL-1β, IL-6, IL-5, and TNF-α) by epithelial cells [86]. In addition to the production of these cytokines, epithelial cells secrete other molecules to recruit immune cells. Recently, cystatin SN has been identified as a key factor in amplifying type 2 inflammation. Cystatin SN promotes eosinophil recruitment and activation through IL-5 [87], upregulates type 2 cytokines, and induces Th2 cell infiltration in the nasal mucosa [88].

### 4.4. Perturbation of Epithelial Homeostasis and Pathogenic Epithelial Stem Cell Memory in CRS

**Figure 2:**
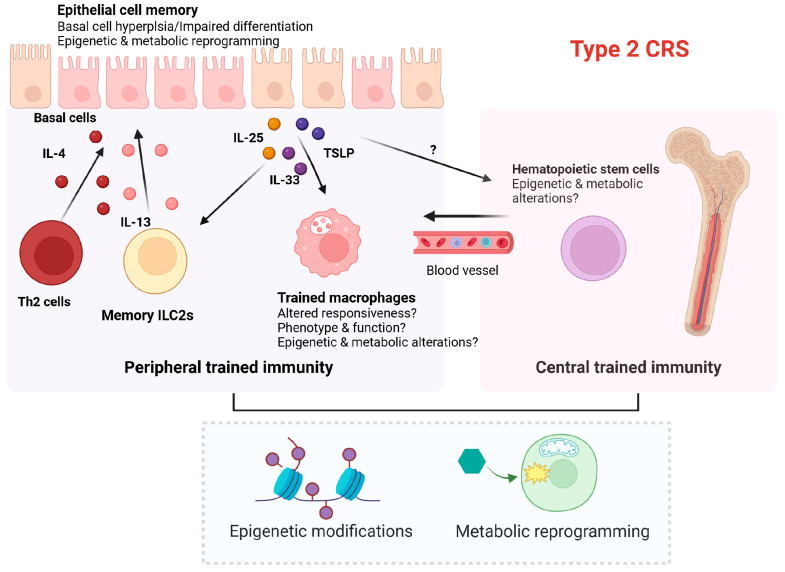
Proposed mechanisms of epithelial cell memory and trained immunity in type 2 CRS. The inflammatory memory of nasal epithelial cells and innate immune cells (such as macrophages and ILC2s) may perpetuate inflammation in the sinonasal mucosa, potentially contributing to the recurrence of type 2 CRS. Long-lasting memory of innate immune cells may be mediated by trained hematopoietic stem cells (central trained immunity). Trained immunity typically involves epigenetic and metabolic reprogramming of innate immune cells. This figure was created in Created with BioRender.

As described above, basal cells are progenitor cells responsible for maintaining homeostasis and integrity of the epithelium [89]. Intriguingly, accumulating evidence has shown hyperplasia of undifferentiated basal cells in CRS (Figure 2). Additionally, restrictions in the differentiation of basal cells into ciliated or secretory cells have been observed in CRS (Figure 2) [90]. Stimulation with type 2 cytokines prevented basal cell differentiation through insulin receptor substrate-dependent signaling in CRS [91], consequently contributing to the loss of ciliated and secretory cells in CRSwNP. Moreover, basal cells from CRSwNP patients exhibited inflammatory memory following exposure to IL-4 and IL-13 (Figure 2) [90].

Because nasal epithelial cells are the first responder to inhaled pathogens, residual pathogenic epithelial cell memory in the sinonasal tissue after treatment, including ESS and biologics therapy, may play a critical role in the initiation of recurrence. It is expected that foreign antigens or triggering factors may perpetuate inflammation by stimulating pathogenic epithelial stem cell memory. Similarly, inflammatory memory in epithelial stem cells was identified in a psoriasis mouse model induced by imiquimod. The chromatin accessibility of inflammation- and hyperproliferation-related genes was increased in skin epithelial stem cells of the imiquimod-treated group compared with the control group [92]. However, knowledge about the mechanisms regulating the pathogenic memory of nasal epithelial stem cells in CRS remains limited. Recent advancements in experimental technologies, including single-cell and spatial multi-omics analyses, may provide deeper insights into epithelial cell biology in CRS. Given that epigenetic imprinting is suggested to confer long-term pathogenic inflammatory memory [92,93,94], elucidating epigenetic alterations in basal cells may facilitate the development of strategies to reverse pathogenic epithelial stem cell memory. Additionally, identifying pathogenic epithelial subsets across the inflammatory endotypes of CRS and comprehensively evaluating these cell subsets at transcriptional and epigenetic levels could help identify molecular targets to modulate pathogenic epithelial memory. Furthermore, considering the growing interest in whether currently available type 2 biologics, such as dupilumab and omalizumab, have disease-modifying effects on CRS, the impact of biologics on pathogenic epithelial memory should be investigated in future studies.

## 5. Possible Roles of Trained Immunity in CRS

### 5.1. Definition and Basic Concept of Trained Immunity

The concept of immunological memory has traditionally been considered a hallmark of adaptive immunity. However, this view of immunological memory cannot explain nonspecific memory responses. Over the past decades, accumulating evidence has shown that innate immune memory mechanisms are evolutionarily conserved [95]. In 2011, Netea et al. introduced the term ‘trained immunity’, suggesting that innate immunity also possesses memory features [96]. Trained immunity is defined as an enhanced immune response of innate immune cells to rechallenges due to immune memory [97]. Trained immunity enables a robust and rapid response to antigen or pathogen restimulation. A well-recognized example of this concept is the Bacillus Calmette–Guérin (BCG) vaccine, which provides cross-protection in immunodeficient mice lacking T and B cells [98]. An increasing number of studies have demonstrated that trained immunity exists in both invertebrates and vertebrates, including mice and humans [90,96,98,99,100,101,102,103].

During the process of trained immunity, primed innate immune cells undergo cellular reprogramming [103]. One of the key molecular mechanisms underlying the induction of trained immunity is epigenetic reprogramming. This process regulates gene transcription through alterations in chromatin structure and DNA methylation. Over the past decade, several studies have established that transcriptional and epigenetic reprogramming form the basis of trained immunity in peripheral innate immune cells and hematopoietic stem cells. Engagement of pattern-recognition receptors activates various intracellular pathways, resulting in the activation of transcription factors such as AP-1 and C/EBPβ, which induce the transcription of genes encoding pro-inflammatory cytokines, including IL-6, IL-1β, and TNF, as well as chemokines. This gene transcription is accompanied by epigenetic changes that increase chromatin accessibility, such as histone methylation and acetylation. The epigenetic regulation of histone modifications, which results in the long-term opening of chromatin, is central to inducing trained immunity [101,104].

Various pathways of metabolic rewiring have been reported to mediate trained immunity. Epigenetic reprogramming is also associated with a metabolic shift in trained macrophages. The induction of trained immunity by stimuli such as BCG vaccines enhances aerobic glycolysis through the activation of the Akt–HIF-1α-mTOR pathway [105]. In parallel, the metabolite mevalonate mediates monocyte training by activating IGF1-R and mTOR, leading to subsequent histone modifications [106]. The accumulation of fumarate, resulting from glutamine replenishment in the TCA cycle, induces innate immune training by inhibiting the lysine demethylase enzyme KDM5, which enables the long-term persistence of H3K4 methylation [107]. Additionally, succinate amplifies IL-1β secretion by bone marrow-derived macrophages via the HIF-1α signaling pathway [108].

Trained immunity can be induced in various innate immune cells from both myeloid (monocytes/macrophages, DCs, neutrophils, microglia) and lymphoid (NK cells and ILCs) cell lineages. Notably, trained immunity in innate immune cells persists for several months [92,98,109]. However, it remains unclear how immune memory persists in circulating innate immune cells given that these cells typically remain in circulation for only a few days. This phenomenon can be explained by the trained immunity of immune progenitor cells in the bone marrow, referred to as ‘central trained immunity’ [103,110]. Innate immune memory of tissue immune cells, a process known as ‘peripheral trained immunity’, has also been demonstrated [103].

### 5.2. Trained Immunity in Chronic Inflammatory Diseases

Given that trained immunity can be induced in several types of innate immune cells, it is suggested that trained immunity plays a crucial role in various diseases. Trained immunity induced by the BCG vaccine mediates heterologous protection against infections. Although the BCG vaccine was developed over a hundred years ago as a tuberculosis vaccine, numerous studies have demonstrated its ability to protect against other infections [93,98]. However, the induction of trained immunity can also have harmful effects. Aberrant induction of trained immunity can detrimentally cause hyperinflammation, resulting in the initiation or progression of chronic inflammatory diseases, such as autoimmune or allergic diseases. Pathological roles of trained immunity have been identified in various chronic inflammatory and autoimmune diseases, including systemic lupus erythematosus [111], lupus nephritis [112], stroke [113], gout [114], and systemic sclerosis [115].

### 5.3. Possible Pathogenic Roles of Trained Immunity in CRS

Several studies have explored the pathogenic roles of trained immunity in asthma, a lower airway disease that shares several characteristics with CRS. Specifically, recent research has shown that macrophages from patients with asthma exhibit an inflammatory transcriptional signature along with metabolic and epigenetic reprogramming. Haimerl et al. suggest that inflammatory training of monocyte-derived macrophages (MDMs) contributes to the pathogenesis of NSAID-exacerbated respiratory disease (NERD) [116], which is a clinical triad of CRSwNP, asthma, and NSAID intolerance. The authors showed that monocytes/macrophages from patients with NERD show alterations in DNA methylation, metabolic profiles, and chemokine expression, suggesting persistent pro-inflammatory activation. Additionally, house dust mite-induced allergic airway inflammation caused inflammatory transcriptional reprogramming and pronounced inflammatory mediator (TNF-α, CCL17, leukotriene, PGE2, and IL-6) responses in monocyte-derived macrophages upon stimulation, mediated by TNF signaling [102]. In addition to macrophages, ILC2s have been reported to be implicated in trained immunity within the context of allergic inflammation. Allergen- and IL-33-experienced ILC2s remain in the lung, draining mediastinal lymph nodes for several months, even after inflammation has been resolved. These IL-33-experienced ILC2s respond more potently upon challenge with unrelated allergens compared with naïve ILC2s and display gene signatures of memory T cells [117]. Repetitive allergenic stress induces a distinct epigenetic landscape and activates a preparedness program in memory ILC2s, leading to type 2 inflammation [118]. Additionally, it appears that hematopoiesis is altered in patients with asthma. Epithelial-derived cytokines affect hematopoietic stem cell activation and differentiation [117,119], as well as basophil hematopoiesis [120], resulting in transcriptional and functional changes in immune cells that promote type 2 inflammation.

Based on studies on asthma, it is highly likely that maladaptive induction of trained immunity in innate immune cells and their bone marrow progenitors may contribute to type 2 CRS (Figure 2). Enhanced inflammatory responses and transcriptional changes in innate immune cells of patients with type 2 CRS may support the involvement of trained immunity in its pathogenesis. However, the mechanisms underlying macrophage reprogramming in type 2 inflammation are not completely understood, and further investigation is required to address the metabolic and epigenetic pathways driving the reprogramming of myeloid progenitors. Additionally, the functional relevance of long-term macrophage reprogramming for chronic inflammation and disease progression in CRS remains to be elucidated. The cellular interactions that maintain and potentiate trained immunity in type 2 inflammation need to be clarified in future studies. Furthermore, whether allergens, epithelial-derived cytokines, or type 2 cytokines can induce central trained immunity by regulating hematopoiesis and hematopoietic stem cells in CRS also requires further investigation. Additionally, increased levels of pro-inflammatory cytokines like IL-1β, TNF, and IL-8 and involvement of macrophages producing IL-1β are key features of non-type 2 CRS. Therefore, it is highly likely that trained immunity is implicated in the pathogenesis of non-type 2 CRS. As non-type 2 CRS is prevalent in East Asian countries [11], both epigenetic and metabolic reprogramming of innate immune cells need to be elucidated in non-type 2 CRS. Mouse models of CRS may provide deeper insights into the duration and underlying mechanisms of trained immunity triggered by allergens or other potential triggering factors such as respiratory viruses. These investigations would not only enhance our understanding of the pathomechanisms but also pave the way for therapeutic targeting of trained immunity in CRS.

## 6. Perspectives on Therapeutic Strategies Targeting Mucosal Inflammatory Memory in CRS

As described above, the treatment of CRS has significantly advanced with the development of ESS and various biologic therapies tailored to specific inflammatory endotypes. However, some patients still experience frequent recurrences even after treatment. Given that CRSwNP is significantly influenced by environmental factors [8,121], recurrence may result from the reactivation of residual inflammatory memory localized in the nasal mucosa, even after partial removal through surgery and the use of biologics such as dupilumab. Therefore, regulating or depleting mucosal inflammatory memory from diseased tissues may be a promising treatment option for the long-term control of and cure for CRS.

**Figure 3:**
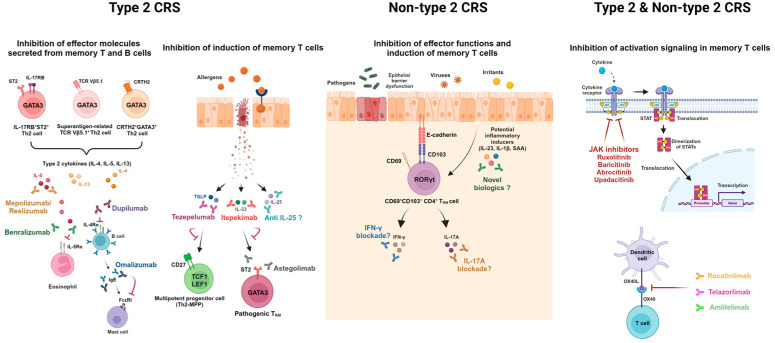
Potential therapeutic strategies targeting pathogenic memory T and B cells for type 2 and non-type 2 CRS. Red arrows indicate inhibitory actions of currently available biologics to treat inflammatory memory. Inhibition of the effector function, inducers, or activation signaling may alleviate immunopathogenesis. This figure was created in Created with BioRender.

Several strategies may be considered for regulating these pathogenic memory cells (Figure 3). First, effector functions of pathogenic memory T and B cells can be inhibited by currently available biologics such as dupilumab, mepolizumab, benralizumab, omalizumab, and infliximab. However, several studies have suggested that current biologics are not sufficient to completely eliminate pathogenic memory cells from diseased tissue. The persistence of pathogenic Th2 effector cells was observed in the skin of patients with clinically resolved atopic dermatitis following treatment with dupilumab [122]. Similarly, pathogenic T_RM_ cells were detected in patients with psoriasis even 6 years after TNF inhibitor treatment [123]. Therefore, inhibition of upstream molecules involved in the induction of pathogenic memory cells may offer more fundamental treatment options for curative intent. Given that nasal pathogenic Th2 cells in patients with CRS expressed high levels of receptors for epithelial-derived cytokines [49,50], these cytokines may play a crucial role in the maintenance and induction of pathogenic memory Th2 cells. In particular, in vitro TSLP stimulation induces the expansion of Th2 progenitor cells and confers resistance to glucocorticoid-induced cell death [52]. Notably, pro-inflammatory cytokines IL-23 and IL-1β are known to be key drivers of the differentiation toward pathogenic Th17 cells during inflammatory conditions [124]. Serum amyloid A also promotes the induction of pathogenic Th17 cells [125]. In this regard, inhibitors or monoclonal antibodies targeting these potential inducers may decrease pathogenic memory T cells in the tissue. Furthermore, inhibiting signaling pathways related to activation or suppressing co-stimulation signals in pathogenic memory T cells may be a promising avenue for developing new treatment strategies. Pathogenic T_RM_ activation can be inhibited by Janus kinase (JAK) inhibitors, such as ruxolitinib, baricitinib, abrocitinib, and upadacitinib, which target signaling pathways activated by IL-4, IL-5, IL-13, and other cytokines [126]. In addition, given that the engagement of OX40–OX40L promotes the expansion of effector T cells and enhances their functions of T cells, targeting this co-stimulation signal may be beneficial. A combination of currently available biologics and novel therapeutics suppressing the induction and activation of memory T cells may offer a powerful treatment option.

**Figure 4:**
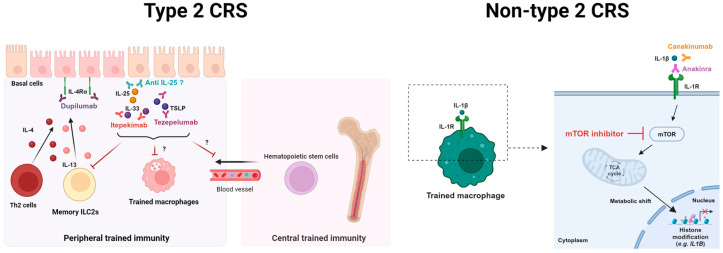
Possible therapeutic strategies for targeting epithelial cell memory and maladaptive trained immunity in type 2 and non-type 2 CRS. Red arrows indicate inhibitory actions of currently available biologics to treat inflammatory memory. In type 2 CRS, duplimab, an IL-4Rα blocking antibody, may inhibit the epigenetic scarring of the nasal epithelium induced by type 2 cytokines from pathogenic memory cells. In-hibition of epithelial-derived cytokines (IL-25, IL-33, TSLP) through itepekimab (anti-IL-33 anti-body) and tezepelumab (anti-TSLP antibody) may suppress the peripheral and central trained immunity. In non-type 2 CRS, the training of IL-1β producing macrophages may be abbrogated through mTOR inhibition. This figure was created in Created with BioRender.

Figure 4 presents the possible therapeutic strategies to abrogate trained immunity of epithelial stem cells and macrophages in type 2 and non-type 2 CRS. Considering that type 2 cytokines induce epigenetic imprinting on the basal cells of the airway epithelium [90], IL-4Rα blocking antibodies such as dupilumab can suppress epigenetic scarring of the epithelium. Although it remains unclear how, nasal epithelial cell-derived cytokines may promote inappropriate training of macrophages and aberrant granulopoiesis in bone marrow hematopoietic stem cells in type 2 CRS [120]. Therefore, itepekimab, a monoclonal antibody targeting IL-33, and tezepelumab, a monoclonal antibody targeting TSLP, may inhibit the induction of central and peripheral trained immunity initiated by epithelial cells. Additionally, IL-1β, a key pro-inflammatory mediator in the non-type 2 immune response, is known to induce trained immunity of macrophages [101]. Canakinumab, an anti-IL-1β agent, and anakinra, an anti-IL-1R agent, inhibit the IL-1β activity of macrophages [127]. Although the factors inducing trained immunity of innate immune cells remain unclear in non-type 2 CRS, these drugs may be potential therapeutic options. Moreover, inhibition of mTOR, a key regulator of metabolic reprogramming in trained macrophages, may be another option for suppressing maladaptive trained immunity in non-type 2 CRS.

## 7. Concluding Remarks

In summary, it is highly likely that the inflammatory memory of adaptive immune cells, innate immune cells, and epithelial cells plays a critical role in initiating recurrence and promoting disease progression in CRS upon exposure to triggering factors. Therefore, targeting the mechanisms or key regulators involved in the inflammatory memory of these cells provides a promising strategy to complement currently available therapies for CRS. We summarized pathogenic alterations of mucosal inflammatory memory in type 2 and non-type 2 CRS (Table 1). Additionally, understanding the cellular network between pathogenic memory cell subsets may enable us to identify novel therapeutic targets. Moreover, investigating the effects of currently approved treatment modalities on memory cells may deepen our understanding of inflammatory memory in humans. However, although this review comprehensively summarizes and discusses the overall concept of mucosal inflammatory memory in the context of CRS, we cannot provide definite alternative approaches or treatments targeting mucosal inflammatory memory due to the limited research available on this topic. Further research should focus on exploring these areas. Additionally, patient-reported outcome measures (PROMs), which are used to measure quality-of-life impairment and symptom severity, are critical for evaluating patients with CRS. Although no studies have directly investigated the association between mucosal inflammatory memory and PROMs, such as the 22-item Sinonasal Outcome Test (SNOT-22), it is plausible that polyp regrowth and increased nasal discharge following reactivation of mucosal inflammatory memory may worsen symptoms such as hyposmia and nasal obstruction, consequently leading to a decline in the quality of life. In this regard, further studies are required to investigate whether the burden of mucosal inflammatory memory may affect PROMs in the long term.

## Figures and Tables

**Table 1:** Summary of pathogenic alterations in each cellular component and evidence supporting mucosal inflammatory memory in CRS.

Pathogenic T Cell Memory		
Type 2	Th2 cells	Express high levels of ST2, IL-17RB, and CRTH2 [46];
		Exhibit high responsiveness to IL-25 and IL-33 [49];
		Produce IL-4, IL-5, IL-13 and amphiregulin upon IL-33 stimulation [44,45,46];
		Superantigen-related TCR Vβ5.1^+^CD4^+^ T cells express high levels of ST2, IL-17RB, and CRTH2 and produce high levels of type 2 cytokines [53].
Non-type 2	Th1/17 cells	CD69^+^CD103^+^CD4^+^ T_RM_ cells are enriched in non-type 2 CRS compared with type 2 CRS and controls [53];
		Express high levels of RORγt and CCR6 and possess robust IFNγ and IL-17A-producing capacities [53].
Pathogenic B cell memory		
Type 2	B cells and ASCs	The frequency of CD19^+^CD27^+^CD38^hi^ plasmablasts is significantly increased in NPs [64]. The number of IgD^+^CD19^+^CD38^bright^ plasmablasts increased in the nasal tissue of CRSsNP [65].
		Expression levels of germline transcripts for IgG, IgA, and IgE are increased in NP tissues [63,67,68].
		Tertiary lymphoid structures or ectopic lymphoid tissues exist in some CRS patients and are associated with local immunoglobulin production [68,70,71].
Non-type 2		Further research is needed.
Pathogenic epithelial stem cell memory		
Type 2		Stimulation with type 2 cytokines prevents the differentiation of basal cells via insulin receptor substrate-dependent signaling in CRS, which, in turn, contributes to the loss of ciliated and secretory cells in CRSwNP [91].
		Basal cells from patients with CRSwNP acquire inflammatory memory after exposure to IL-4 and IL-13 [90].
Non-type 2		Further research is needed.
Possible pathogenic roles of trained immunity		
Type 2	MDMs from patients with NERD	Exhibit an overall reduction in DNA methylation, aberrant metabolic profiles, and increased expression of chemokines, suggesting persistent pro-inflammatory activation [116].
	MDMs from HDM-allergic patients and HDM-induced AAI mice	Exhibit inflammatory transcriptional reprogramming and excessive inflammatory mediator (TNF-α, CCL17, leukotriene, PGE2, and IL-6) responses via TNF signaling [102].
	Allergen- and IL-33-experienced ILC2s from mice	Persist in the lung and draining mediastinal lymph nodes for several months, even after inflammation has resolved [128];
		Respond more potently upon challenge with unrelated allergens compared to naïve ILC2s and display gene signatures of memory T cells [128].
	Hematopoietic stem cells from mice	Epithelial-derived cytokines affect hematopoietic stem cell activation and differentiation, as well as basophil hematopoiesis, resulting in transcriptional and functional changes in immune cells that promote type 2 inflammation [117,119,120].
Non-type 2	IL-1β-producing macrophages	Increased levels of pro-inflammatory cytokines, such as IL-1β, TNF, and IL-8, and involvement of IL-1β-producing macrophages are key features of non-type 2 CRS [129,130].

AAI, allergic airway inflammation; ASCs, antibody-secreting cells; HDM, house dust mite; MDM, monocyte-derived macrophage; NERD, NSAID-exacerbated respiratory disease.

## Data Availability

No new data were created or analyzed in this study. Data sharing is not applicable to this article.
